# Liver abscessation and multiple septic pulmonary emboli associated with Lemierre’s syndrome: a case report

**DOI:** 10.1186/s13104-015-1028-9

**Published:** 2015-03-03

**Authors:** Yuichi Takano, Kenichiro Fukuda, Hiromi Takayasu, Kazuki Shinmura, Go Koizumi, Masahiro Sasai, Yoshikuni Nagayama, Michiari Kawamo, Tomohiro Yasuda, Kazumasa Watanabe, Jun Sasaki, Munetaka Hayashi, Eiichi Yamamura, Naotaka Maruoka, Masatsugu Nagahama, Hiroshi Takahashi

**Affiliations:** Department of Emergency and Critical Care Medicine, Showa University Fujigaoka Hospital, 1-30 Fujigaoka, Aoba-ku, Yokohama-shi, Kanagawa 227-8501 Japan; Division of Gastroenterology, Department of Internal Medicine, Showa University Fujigaoka Hospital, Yokohama, Kanagawa Japan

**Keywords:** Lemierre’s syndrome, Liver abscess, *Fusobacterium necrophorum*, Septic pulmonary emboli

## Abstract

**Background:**

In Lemierre’s syndrome, patients first exhibit pharyngitis and peritonsillar abscessation, followed by the development of anaerobic bacterial (usually *Fusobacterium necrophorum*) septicemia and metastatic infections throughout the body. However, these infections rarely affect the liver. We describe a case of Lemierre’s syndrome, in which the first disease manifestation was liver abscess, for drawing attention of emergency physicians to this rare but fatal disease.

**Case presentation:**

A 28-year-old Asian ethnicity Filipino male, who was previously healthy, entered the emergency department presenting with fever and pharyngeal pain that had persisted for 5 days. Contrast-enhanced abdominal computed tomography revealed a 3-cm area of low density in segment 6 of the liver, consistent with an abscess. Chest computed tomography also revealed that multiple nodes in both lungs were enlarged, and septic emboli were suspected. The patient was hospitalized and antibiotic treatment was initiated. On hospital day 6, blood culture results confirmed *Fusobacterium necrophorum* septicemia. The patient was diagnosed with Lemierre’s syndrome, as pharyngitis developed into bacteremia associated with hepatic and pulmonary lesions. The patient’s condition improved with antibiotics and he was discharged following three weeks of treatment in the hospital.

**Conclusion:**

With the widespread use of antibiotics, Lemierre’s syndrome is rarely encountered anymore, but it can be fatal if not properly diagnosed. It is a crucial differential diagnosis in young patients exhibiting septicemia or multiple metastatic infection of unknown origin.

## Background

Lemierre’s syndrome (LS) usually first presents with pharyngitis and peritonsillar abscessation, which are followed by the development of anaerobic bacterial (usually *Fusobacterium necrophorum*) septicemia, internal jugular vein (IJV) thrombi, and multiple septic emboli [1]. LS leads to metastatic infections in organs throughout the body, but rarely causes liver abscess formation (only 2% to 4% of cases) [2]. Although the reason is unknown, this disease often occurs in healthy, young individuals. Because antibiotic usage has become routine, LS has become a relatively uncommon disease, but it can be fatal if not properly diagnosed. Here we report a case of LS in which the first manifestation was liver abscess, along with a discussion of the literature.

## Case presentation

A previously healthy 28-year-old Asian ethnicity Filipino male entered our emergency department presenting mainly with complaints of fever, pharyngeal pain, and headache that had lasted 5 days. His vital signs were as follows: body temperature, 39.3°C; blood pressure, 96/50 mmHg; pulse rate, 136 beats/min; respiration rate, 28 breaths/min; saturation pulse oxygen (SpO_2_), 97% (room air). Mild rubefaction of the pharynx was observed, but tonsillar swelling and excessive tongue fur were not observed. Abdominal examination revealed no pain in response to pressure and diminished bowel sounds.

Laboratory tests revealed that transaminase was normal (Glutamic Oxaloacetic Transaminase, GOT = 35 U/l, normal = 10–40; Glutamic pyruvic transaminase, GPT = 24 U/l, normal = 5–45) and biliary enzymes were slightly elevated (Alkaline phosphatase, ALP = 499 U/l, normal = 110–360; Gamma-glutamyl transpeptidase, γ-GTP = 108 U/l, normal < 45). White blood cell and platelet counts were decreased [3400/μl (normal = 3500–9100) and 30000/μl (normal = 130000–369000), respectively], and septicemia was suspected. C-reactive protein (CRP) was markedly elevated (28.67 mg/dl, normal = 0–0.3). A diagnosis of systemic inflammatory response syndrome (SIRS) was indicated as the patient fulfilled all four diagnostic criteria for SIRS.

**Figure 1:**
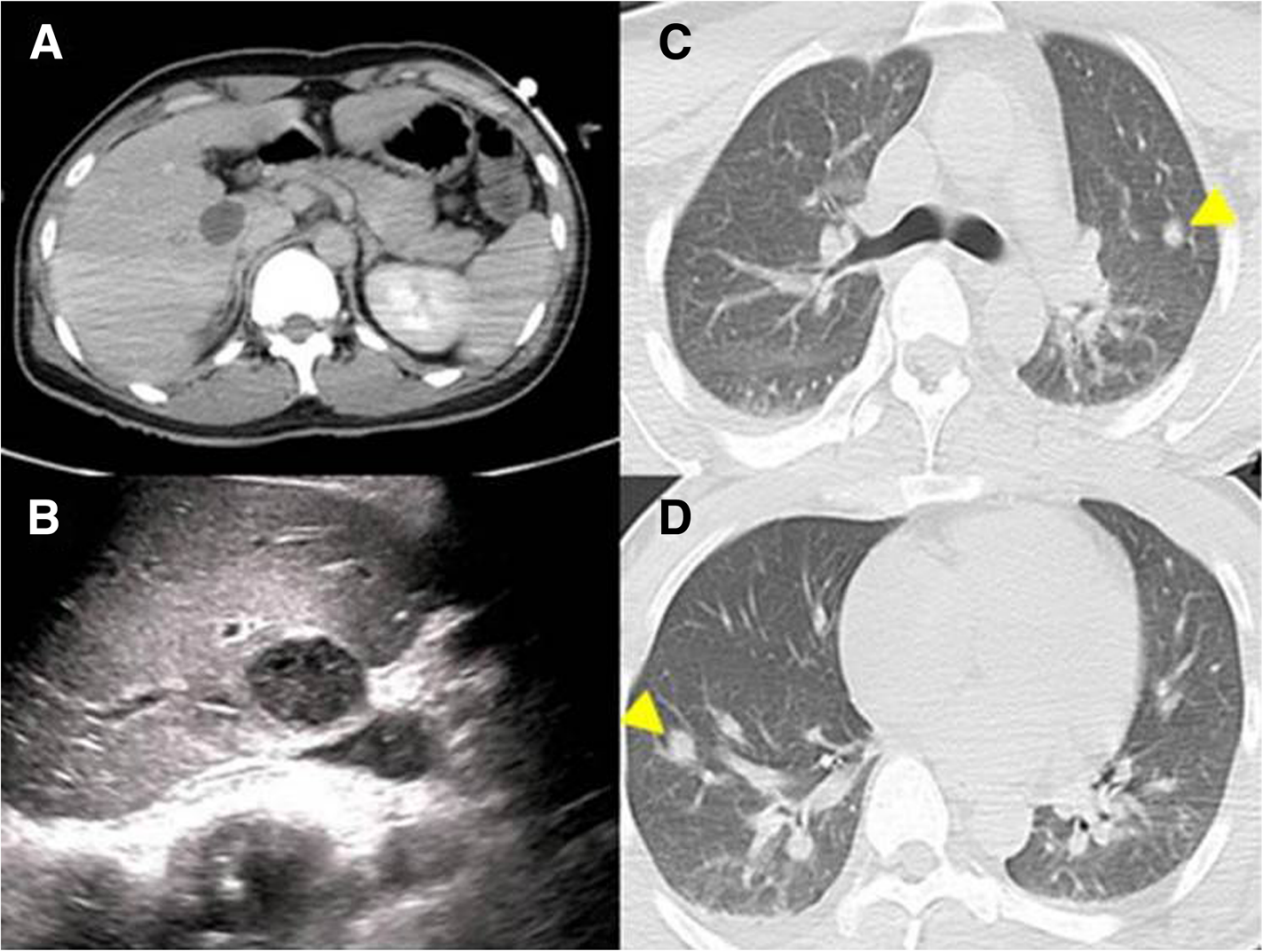
**Images of computed tomography and abdominal ultrasound. A**: Contrast computed tomography revealed a 3-cm low density area in segment 6 of the liver. Slight ring-shaped contrast effects were observed in the lesion area. Liver abscess was diagnosed. **B**: Abdominal ultrasound revealed an oval-shaped, hypoechoic lesion with clear borders in segment 6 of the liver. **C**, **D**: Multiple nodes were observed in the left superior lobe and right inferior lobe of the lung (arrowheads). Septic emboli were suspected. These nodes completely disappeared with treatment.

Abdominal Computed tomography (CT) revealed a 3-cm area of low density in segment 6 (S6) of the liver. Slight ring-shaped contrast effects were observed in the lesion area and a liver abscess was diagnosed (Figure 1A). Abdominal ultrasound confirmed a 3-cm hypoechoic lesion in S6 (Figure 1B). Additional CT identified multiple nodes in both lungs, and septic emboli were suspected (Figure 1C and D). The patient was hospitalized with a diagnosis of liver abscess and septicemia, and treatment with antibiotics was initiated.

On day 6 of hospitalization, *Fusobacterium necrophorum* was detected in two sets of blood cultures for anaerobes taken at the time of admission. Consistent with the diagnostic criteria outlined by Riordan *et al*., the patient was diagnosed with LS, as pharyngitis developed into *Fusobacterium necrophorum* bacteremia and lesions subsequently developed in the liver and lungs.

Echocardiography did not reveal any verrucae, so infective endocarditis was ruled out. No clear thrombi were identified in the IJV using ultrasound. Lower gastrointestinal (GI) endoscopy was performed, but the findings were normal. The patient tested negative for serum amoebic dysentery and Human Immunodeficiency Virus (HIV) antibodies. Hemoglobin A1c (HbA1c) level was also normal (5.5%, normal = 4.6–6.2), as was glucose tolerance. A dental examination was performed to test for intraoral infection, but no abnormalities were found.

Intravenous ceftriaxone (CTRX) was administered at 4 g/day for 6 days. After detection of *Fusobacterium necrophorum* in the blood cultures, we changed antibiotics to intravenous meropenem (MEPM) at 2 g/day for 14 days. The patient responded favorably to this drug and was discharged from the hospital three weeks later. Drainage of the liver abscess was not performed.

## Discussion

LS was identified by André Lemierre in 1936. In this syndrome, bacteremia caused by anaerobic bacteria develops from pharyngitis or peritonsillar abscess, after which thrombophlebitis of the IJV occurs and metastatic infections develop throughout the body, particularly in the lungs [1]. The syndrome often affects healthy, young individuals, and the most common causative agent is *Fusobacterium necrophorum. Fusobacterium* species are obligate, anaerobic, non-sporous gram-negative bacteria that are present in the mouth, throat, and digestive tract of humans. *Fusobacterium necrophorum* and *Fusobacterium nucleatum* are pathogenic to humans, with the former considered to have stronger pathogenicity [2,3].

LS was first reported as a fatal disease that mainly occurred in Europe, but it has become rare in modern times, and diagnosis is often difficult [2]. The mortality rate has improved with the use of antibiotics, but it has still been reported as being as high as 10% [2]. Initially, we treated our patient for liver abscess and septicemia of unknown cause, but the detection of *Fusobacterium necrophorum* in blood cultures allowed us to reach a definitive diagnosis of LS.

Chirinos *et al*. reported that in 69.7% of LS cases, the first clue for its diagnosis is the detection of *Fusobacterium necrophorum* in blood culture, rather than clinical signs or symptoms [3]. This suggests that many clinicians are unable to diagnose LS based on early clinical features.

The criteria for the diagnosis of LS are not clearly defined. Riordan *et al*. [2] proposed the following three diagnostic criteria on the basis of their examination of many LS cases: (i) history of throat angina in the preceding 4 weeks or compatible clinical findings, (ii) history of metastatic septic emboli, and (iii) evidence of IJV thrombophlebitis or isolation of *Fusobacterium necrophorum* or *Fusobacterium* species from blood cultures or a normal sterile site. Our patient fulfilled all three of these criteria, and therefore, exhibited classic LS.

LS leads to metastatic infections in organs throughout the body, but rarely causes liver abscess formation [2]. Iwasaki *et al*. [4] reviewed 13 cases of LS complicated by liver abscesses. Patients were generally young, with a mean age of 21.7 years. Eight of these patients exhibited multiple abscesses, 4 exhibited single abscesses (not specified in 1 case), and 8 underwent drainage.

No standards for antibiotic selection exist for LS owing to its rarity. Previous studies have reported the efficacy of penicillin, cephem, macrolide, and metronidazole. However, caution must be exercised when selecting antibiotics for the treatment of LS, as *Fusobacterium necrophorum* exhibits resistance as high as 22% to 60% against penicillin and macrolides [5]. Iwasaki *et al.* reported treating a case of LS accompanied by a liver abscess with a single agent (carbapenem) [4]. In addition, antibiotic administration treatment periods of various lengths have been reported across cases, with a range of 2 to 12 weeks.

## Conclusion

In conclusion, LS is a rare disease that is often difficult to diagnose, and it can be fatal if not properly diagnosed and treated. When septicemia or multiple infectious lesions of unknown origin are observed in a previously healthy, young individual, a differential diagnosis of LS should always be considered.

## Consent

Written informed consent was obtained from the patients for the publication of this Case Report and any accompanying images. A copy of the written consent is available for review by the Editor-in-Chief of this journal.

## Abbreviations

LS: Lemierre’s syndrome
IJV: Internal jugular vein
SpO_2_: Saturation pulse oxygen
GOT: Glutamic oxaloacetic transaminase
GPT: Glutamic pyruvic transaminase
ALP: Alkaline phosphatase
γ-GTP: Gamma-glutamyl transpeptidase
CRP: C-reactive protein
SIRS: Systemic inflammatory response syndrome
Lower GI endoscopy: Lower gastrointestinal endoscopy
HIV: Human immunodeficiency virus
HbA1c: Hehmoglobin A1c
CTRX: Ceftriaxone
MEPM: Meropenem
